# A Case of Candidal Infection of Large Biloma After CT-Guided Liver Biopsy Confirming Autoimmune Hepatitis

**DOI:** 10.7759/cureus.11059

**Published:** 2020-10-20

**Authors:** Sindhura Kolli, Vahe Shahnazarian, Harini Gurram, Madhavi Reddy, Krishna Gurram

**Affiliations:** 1 Internal Medicine, New York University (NYU) Langone Hospital, New York, USA; 2 Gastroenterology and Hepatology, The Brooklyn Hospital Center, Affiliate of the Icahn School of Medicine Mount Sinai, Brooklyn, USA; 3 Internal Medicine, Northwestern University Feinberg School of Medicine, Chicago, USA; 4 Gastroenterology and Hepatology, Elmhurst Hospital, Affiliate of Icahn School of Medicine Mount Sinai, Queens, USA

**Keywords:** biloma, candida, fungal, autoimmune hepatitis

## Abstract

Bilomas are unexpected post-procedural complications of cholecystectomies, hepatic resections, endoscopic retrograde cholangiopancreatography, and-rarely-liver biopsies. While uncommon, this should remain high on the differential in a patient presenting with sudden postop sepsis even in the absence of significant liver injury. Diagnosis involves coordination between interventional radiology, gastroenterology, and internal medicine. This involves a precise clinical history, invasive radiological techniques, and advanced interventional endoscopic solutions for diagnosis and treatment. This is a case of a biloma that occurred as a consequence of a post-hepatic biopsy and resulted in fungal infection, rather than its more common bacterial counterpart. Due to the unusual circumstances, a more circuitous route to the correct diagnosis and treatment was undertaken.

## Introduction

When faced with sepsis in a patient who recently underwent an invasive procedure near the biliary system, it is essential to exclude biloma even when it is not high on the differential. While mostly asymptomatic and benign, when superimposed with an infection, bilomas can lead to a cascade of complications requiring rapid diagnosis and treatment with either percutaneous drainage or more invasive procedures such as endoscopic retrograde cholangiopancreatography (ERCP) or hepatic resection. This is a case of a biloma that occurred after a liver biopsy. However, instead of becoming infected with more commonly introduced bacteria, the cultures paradoxically demonstrated a fungal infection. Herein, we expand on the known epidemiology, clinical presentation, and the standard methods of diagnosis and treatment of bilomas. Additionally, we use this case to bring light to an uncommon occurrence while proposing a diagnostic and treatment algorithm for intrahepatic bilomas.

## Case presentation

**Figure 1:**
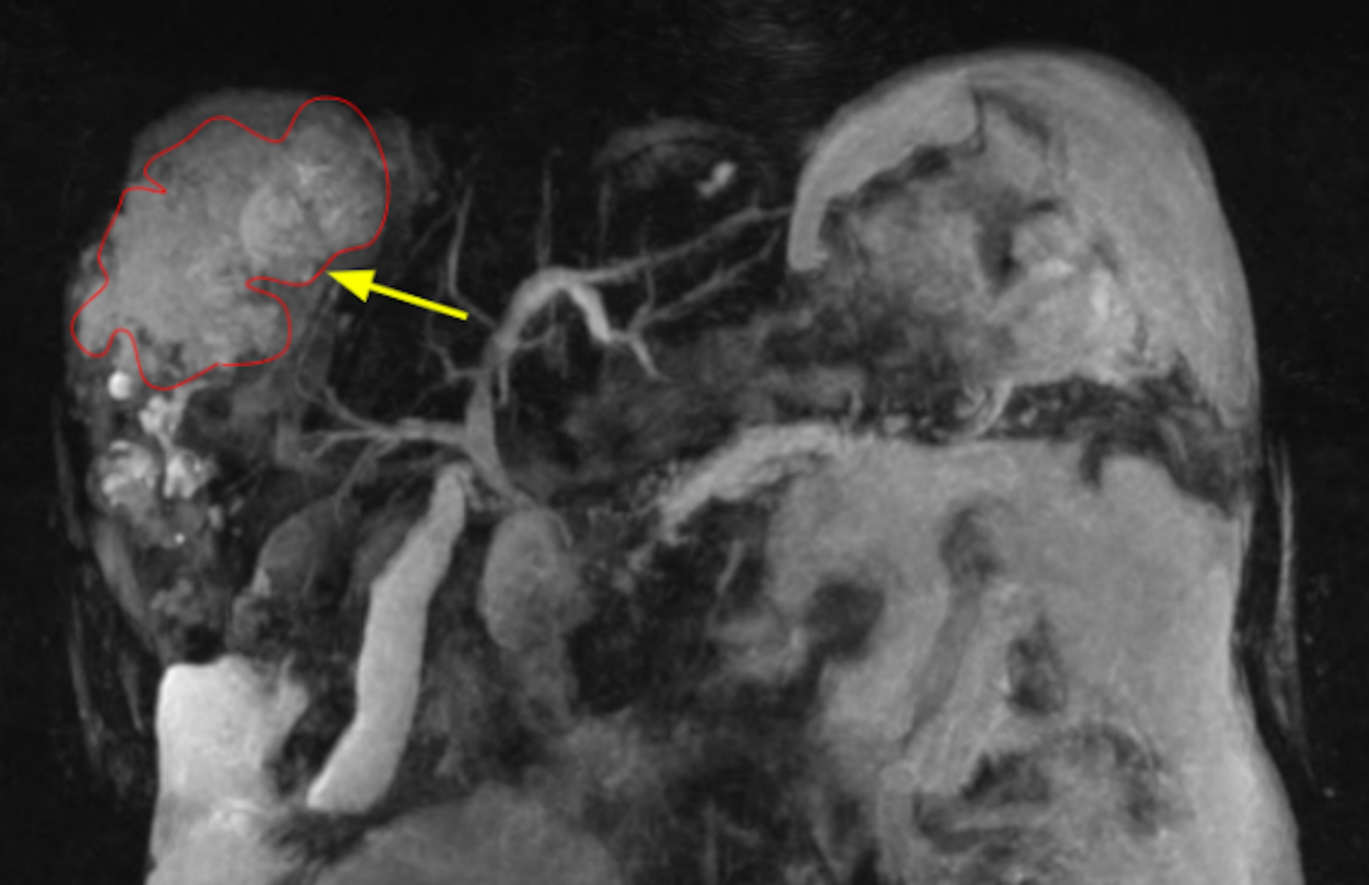
Arrow - large biloma collection involving a large portion of the right lobe of the liver; Red line - biloma outline

A 64-year-old male with a medical history of alcohol abuse, chronic pancreatitis, and recent computed tomography (CT)-guided liver biopsy (performed a week prior to admission, confirming autoimmune hepatitis) presented to the emergency department with lethargy, dizziness, anorexia, and intermittent diffuse abdominal pain. Vitals were: 100.1°F temperature, heart rate 102 beats per minute, and blood pressure 93/60 mmHg. Abnormal labs included neutrophil-predominant leukocytosis of 22.8 cells/L (4.5 to 11.0 × 109/L), alanine transaminase (ALT) of 111 u/L (7-56 units/liter), aspartate transaminase (AST) of 134 u/L (10-40 units/liters), and total bilirubin of 16 mg/dL (0.1-1.2 mg/dL), with 14 mg/dL (0.1-0.3 mg/dL) of it direct bilirubin. A CT showed multiple lobulated cystic lesions in the right hepatic lobe communicating with the intrahepatic biliary ducts indicating a biloma, which was later confirmed by magnetic resonance imaging (MRI) with contrast of the abdomen and pelvis (Figure 1).

**Figure 2:**
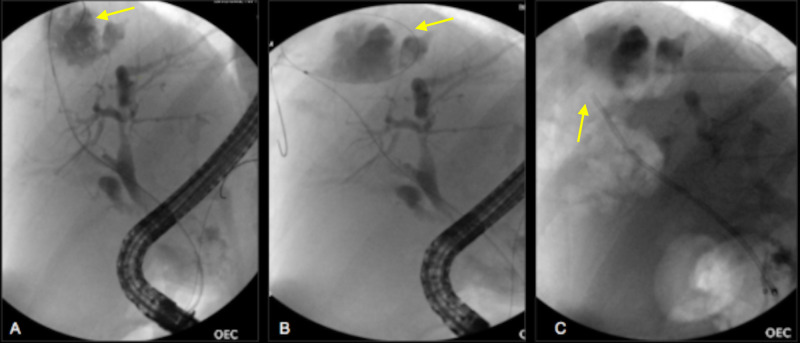
Fluoroscopic images in sequential order 2A. Contrast injection into the biloma. 2B. Wire coiled in the large biloma. 2C. Air in the biloma cavity after placement of plastic stent with transpapillary drainage

Piperacillin-tazobactam was empirically started. During the ERCP, fluoroscopic images demonstrated a large biloma in segments seven and eight of the liver (Figure 2A-2C). A 7 French (Fr) x 12 centimeter (cm) stent was placed and the biloma was drained.

**Figure 3:**
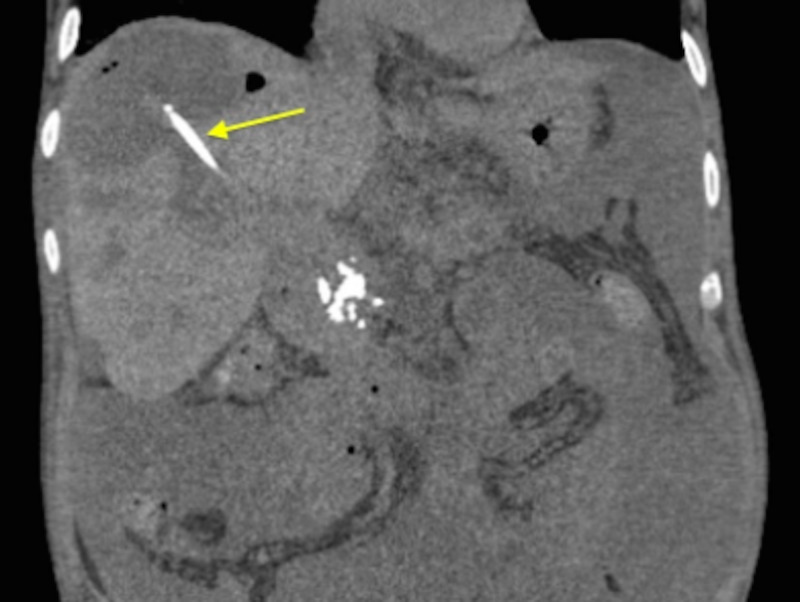
Post-procedural CT of the abdomen with an arrow demonstrating the stent within the biloma

Culture of the biloma fluid revealed candida and numerous neutrophils, prompting the addition of fluconazole. A postoperative CT of the abdomen showed no change in the size of the biloma (Figure 3).

**Figure 4:**
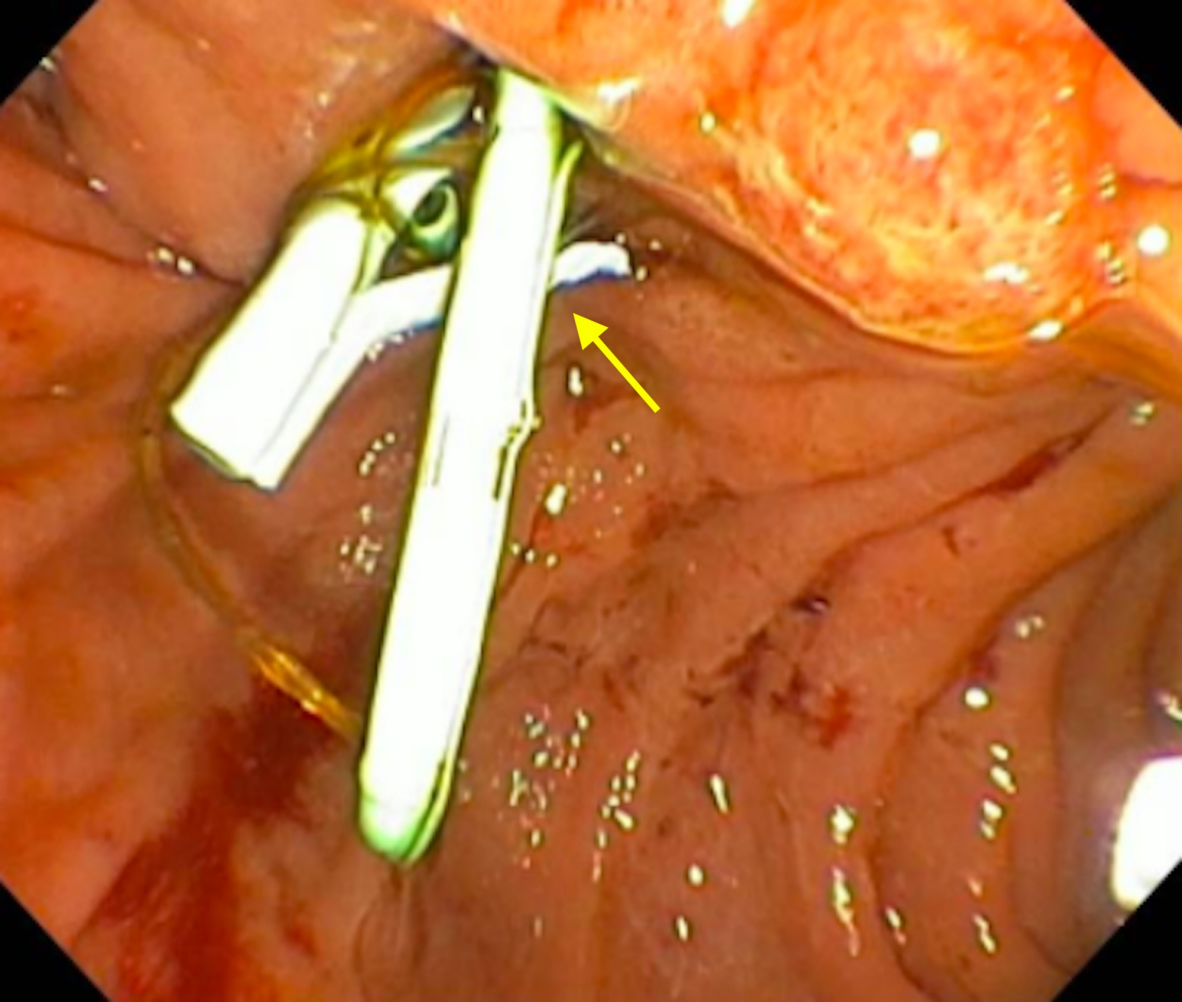
Endoscopic image of the stent in place providing biloma drainage

The patient left against medical advice but returned with similar complaints two weeks later. While vitals were within normal limits, abnormal labs were similar to the previous admission. A repeat ERCP showed the previous stent was patent, but there was still a large collection and biliary sludge within segments seven and eight of the liver. The previous stent was then replaced with a 10 Fr x 12 cm (Figure 4). Having symptomatically improved, the patient again left against medical advice and was lost to follow-up.

## Discussion

Bilomas are collections of bile, either intra- or extrahepatic, with or without a capsule. Their incidence in men and women is equal and occurs in the seventh and eighth decades of life, within a week of the inciting event. They are rare and occur due to disruptions within the biliary system. Disruptions or injuries are often secondary to laparoscopic cholecystectomies, post-Billroth II procedures, poor revision to the cystic duct stump, or puncturing the accessory ducts of Luschka. Other causes include blunt trauma to the abdomen, radiofrequency ablation or resection of hepatocellular carcinoma, and, rarely, spontaneous formation of bilomas secondary to an obstruction or rupture within the biliary system (Table 1) [1-8].

**Table 1:** Etiology resulting in the formation of bilomas ERCP: endoscopic retrograde cholangiopancreatography Sources: [4-8]

Causes of Biloma
Most common causes
Laparoscopic cholecystectomies:
· Poor revision to the cystic duct stump
· Puncturing the accessory ducts of Luschka
Billroth II procedures
Other causes
Liver biopsy
Blunt abdominal trauma
Radiofrequency ablation
Resection of hepatocellular carcinoma
Spontaneous biliary system obstruction or rupture
ERCP
Percutaneous ethanol injection

In slow chronic leaks, a reactive process of inflammation and fibrosis creates a capsule supported by adhesions from the surrounding mesentery and omentum to encapsulate the bile. If the bile leak is more acute, resultant peritonitis and sepsis can occur. Secondary infections of the bile within the biloma are another common sequelae. Common organisms include Enterobacteriaceae (43.0%), Enterococcus (32.2%), and, less commonly, Candida (9.1%) [9]. Clinical symptoms range from abdominal fullness, dull right upper quadrant pain to nausea, vomiting, fever, and gram-negative sepsis [1].

Diagnosis begins non-invasively, with an ultrasound (US) showing cysts, anechoic collections, or septated loculations (indicating infection) [10]. Aspiration of the noted collection(s) is needed for confirmation. CT scans are an option but have low specificity and are ineffective in ruling out differentials such as hematomas, seromas, or pseudocysts. The gold standard continues to be a hepatobiliary cholescintigraphy with a Tc-99m-iminodiacetic acid chelate complex as a tracer, which can detect active or sealed leaks and guide treatment. Its limitations in identifying the exact point of leak due to poor demonstration of detailed anatomy can be circumvented with the concurrent use of a hybrid single-photon emission computed tomography (SPECT/CT) or magnetic resonance cholangiopancreatography (MRCP), which provide a more detailed depiction of the origin of leak showing points of connection between the biloma and the biliary system with an exactness of 79%-85% [11]. More invasive methods, such as ERCP, can also identify and simultaneously treat bilomas [1-3]. They must be distinguished from differentials such as pseudocysts, seromas, abscess, or hematomas [12].

**Figure 5:**
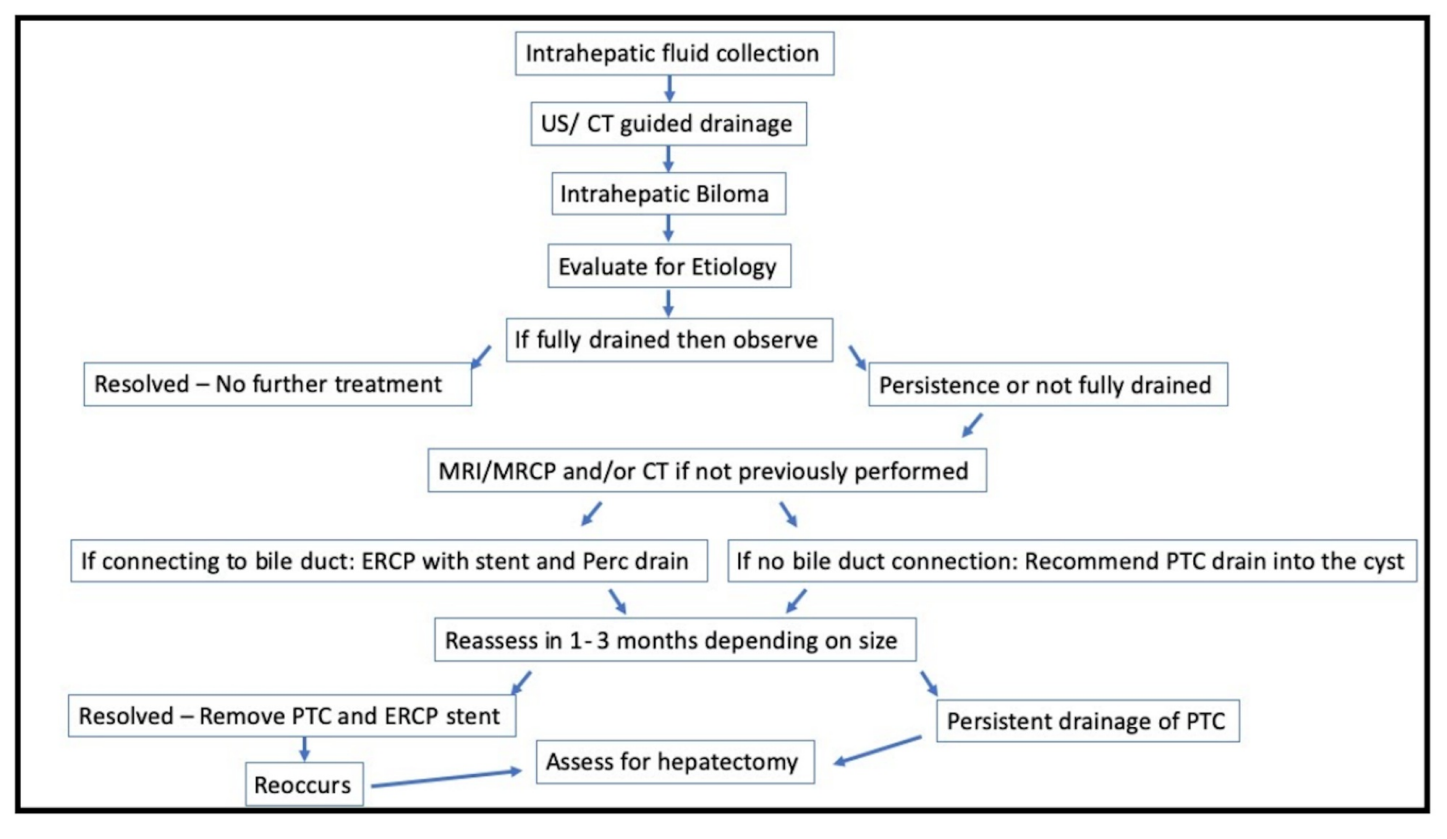
Proposed algorithm for an intrahepatic biloma

When asymptomatic and small, bilomas can be monitored with frequent surveillance. Only when they leak and create symptoms, or become infected, is intervention indicated. Options for treatment are based upon the patient’s overall clinical symptoms. Proceeding directly to an ERCP is possible if adequate mapping of the biloma and intraductal connections is obtained by an MRCP. However, if there are scant or nil biloma duct communications, a percutaneous transhepatic cholangiography (PTC) is done beforehand to illuminate the biliary anatomy before intervention to precisely identify leaks. Then, either a percutaneous transhepatic biliary drainage (PTBD) with an external drain or stricture dilatation or percutaneous drainage are options. An ultrasound (US) or CT afterwards is necessary to confirm that the biloma had drained. If still persistent, an ERCP to decompress, drain, and stent the biloma in combination with a PTC or surgical resection of the liver with biloma are options once the patient is no longer septic (Figure 5) [1-3].

## Conclusions

When asymptomatic and of small size, bilomas do not need to be disturbed. However, once infected or symptomatic, they require immediate diagnosis and intervention. Mapping of the communication prior to an ERCP decompression and confirmatory imaging postoperatively is ideal. Cultures are recommended instead of empirical antibacterials, given that, in this case, the source was fungal and would have prolonged symptoms without resolution. When bilomas are recurrent, a repeat ERCP with a stent might be necessitated, thus making postoperative imaging key to the treatment course.
